# Impact of the COVID‐19 pandemic on colorectal cancer surgery in Japan: Clinical Study Group of Osaka University―A multicenter retrospective study

**DOI:** 10.1002/ags3.12616

**Published:** 2022-08-20

**Authors:** Masaaki Miyo, Tsunekazu Mizushima, Junichi Nishimura, Taishi Hata, Mitsuyoshi Tei, Yuichiro Miyake, Yoshinori Kagawa, Shingo Noura, Masakazu Ikenaga, Katsuki Danno, Atsuhiro Ogawa, Yoshinao Chinen, Tsuyoshi Hata, Norikatsu Miyoshi, Hidekazu Takahashi, Mamoru Uemura, Hirofumi Yamamoto, Kohei Murata, Yuichiro Doki, Hidetoshi Eguchi, Clinical Study Group of Osaka University Colorectal Group

**Affiliations:** ^1^ Department of Surgery National Hospital Organization Osaka National Hospital Osaka Japan; ^2^ Department of Surgery Osaka Police Hospital Osaka Japan; ^3^ Department of Gastroenterological Surgery Osaka International Cancer Institute Osaka Japan; ^4^ Department of Surgery Kansai Rosai Hospital Amagasaki Japan; ^5^ Department of Surgery Osaka Rosai Hospital Sakai Japan; ^6^ Department of Surgery Sakai City Medical Center Sakai Japan; ^7^ Department of Gastroenterological Surgery Osaka General Medical Center Osaka Japan; ^8^ Department of Gastroenterological Surgery Toyonaka Municipal Hospital Toyonaka Japan; ^9^ Department of Gastroenterological Surgery Higashiosaka City Medical Center Higashiosaka Japan; ^10^ Department of Surgery Minoh City Hospital Mino Japan; ^11^ Department of Surgery Tanemulti‐solution General Hospital Osaka Japan; ^12^ Department of Gastroenterological Surgery Graduate School of Medicine, Osaka University Suita Japan

**Keywords:** colonoscopy, colorectal cancer, COVID‐19, pandemic, screening, surgery

## Abstract

**Aim:**

Due to the overwhelming spread of SARS‐CoV‐2 and its disruption of the healthcare system, delays and reduced numbers were reported for colorectal cancer screening, colonoscopies, and surgery during the COVID‐19 pandemic. This multicenter retrospective study investigated the still poorly understood impact of the COVID‐19 pandemic on colorectal cancer treatment in Japan.

**Methods:**

This study was organized by the Clinical Study Group of Osaka University, which comprised 32 major institutions in Osaka. We retrospectively analyzed the number of surgeries and colonoscopies performed and the characteristics of patients who underwent surgery for colorectal cancer between March 2019 and February 2021. We compared data collected before and during the COVID‐19 pandemic. We also assessed the methods used for detecting colorectal cancer, including fecal occult blood test, abdominal symptoms, and anemia.

**Results:**

The COVID‐19 pandemic caused reductions in the annual numbers of surgeries (3569 vs 3198) and colonoscopies (67 622 vs 58 183) performed in the 2020 fiscal year, compared to the 2019 fiscal year. During the COVID‐19 pandemic, a significantly lower proportion of patients were treated for clinical stages ≤I (24.2% vs 26.9%; *P* = .011), compared to the proportion treated before the pandemic. Fecal occult blood tests for detecting colorectal cancer were used significantly less frequently during the COVID‐19 pandemic (26.2% vs 29.6%; *P* = .002). These trends were more significant in larger institutions.

**Conclusion:**

The COVID‐19 pandemic reduced the number of colonoscopies and surgeries performed for colorectal cancer and hindered the detection of asymptomatic early‐stage cancers, and its impact varied by hospital size.

## Graphical abstract

This study aimed to investigate the impact of the COVID‐19 pandemic on colorectal cancer treatment in Japan, that was not fully understood. The COVID‐19 pandemic has reduced the number of colonoscopies and surgeries for colorectal cancer and hindered the detection of asymptomatic early‐stage cancers. " cd_value_code="text

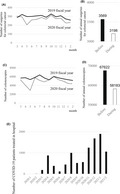

## 1 INTRODUCTION

In December 2019, the first case of a new type of infection caused by a new coronavirus variant, known as severe acute respiratory syndrome coronavirus‐2 (SARS‐CoV‐2) was reported in Wuhan, China. Subsequently, the disease spread rapidly around the world, which led to the COVID‐19 pandemic.^1^, ^2^ In response to the overwhelming spread of the SARS‐CoV‐2 outbreak and its disruption of healthcare systems, many governments worldwide imposed lockdowns or declared states of emergency to prevent its spread.^3^, ^4^ In Japan, the first case of COVID‐19 was confirmed in January 2020. That case was followed by outbreaks on board the Diamond Princess cruise ship, in February 2020, which resulted in 696 cases.^5^ As the number of infections increased, on April 7, 2020, the Japanese government declared states of emergency for Tokyo, Kanagawa, Saitama, Chiba, Osaka, and Hyogo. Subsequently, the state of emergency was extended to all prefectures on April 16, 2020. As of December 2021, more than 1.7 million people had been infected, and there is no sign of the pandemic ending.

The basic routes of SARS‐CoV‐2 transmission were thought to be droplet and contact infections. However, the World Health Organization (WHO) and the Centers for Disease Control and Prevention (CDC) have suggested the possibility that infectious particles could be airborne, which could increase the risk of SARS‐CoV‐2 spreading during endoscopic examinations.^6^, ^7^ About 20%–50% of infected patients are asymptomatic, but nevertheless they can transmit SARS‐CoV‐2 to others.^8^, ^9^ The Japan Gastroenterological Endoscopy Society recommended that elective nonurgent endoscopic procedures should be delayed during a declared state of emergency, as recommended by the New York Society for Gastrointestinal Endoscopy guidelines.^10^, ^11^ Consequently, only one‐quarter of the number of people screened the previous year in Japan were screened in April and May 2020. Moreover, an international survey of 252 centers in 55 countries indicated that the number of colonoscopies performed had decreased by 85%, due to the COVID‐19 pandemic.^12^, ^13^ Currently, there is concern that a decline in the number of screening and colonoscopy examinations performed during the COVID‐19 pandemic may have resulted in delays in detecting early‐stage colorectal cancer, which could lead to an increase in the rate of advanced colorectal cancers. Therefore, the present multicenter retrospective cohort study investigated the impact of the COVID‐19 pandemic on colorectal cancer treatments, including the stage at diagnosis, in Japan.

## 2 MATERIALS AND METHODS

### 2.1 Study design and data extraction

This study was organized by the Clinical Study Group of the Osaka University, Colorectal Group, which comprised 32 institutions affiliated with the Department of Gastroenterological Surgery, Graduate School of Medicine, Osaka University. We retrospectively analyzed a cohort of patients who had undergone surgery for colorectal cancer between March 2019 and February 2021. Medical and pathology reports from all 32 institutions were reviewed to extract data regarding diverse clinicopathological parameters, including sex, age, body mass index (BMI), American Society of Anesthesiologists‐physical status (ASA‐PS), tumor location, surgical approach, surgical procedure, depth of tumor invasion, lymph node metastasis, distant metastasis, and stage, based on the Japanese Classification of Colorectal, Appendiceal, and Anal Carcinoma.^14^ We also recorded the methods used for detecting colorectal cancer, including a fecal occult blood test, abdominal symptoms, anemia, and accidental detection. In addition, we collected information on the 32 institutions, including the number of beds, the annual number of colorectal cancer surgeries and colonoscopies, the response to COVID‐19, and the restrictions placed on gastrointestinal surgery. Patients with missing data, locally resected colorectal cancer, and colorectal cancer recurrences were excluded from the analysis. The study was approved by the Institutional Review Board for Studies in Humans at Osaka University (approval number: 20527) and at each institution. Patients could opt out of the study via the hospital website.

### 2.2 Outcomes

The period between March 2019 and February 2020 was defined as the period before the COVID‐19 pandemic, and the period between March 2020 and February 2021 was defined as the period during the COVID‐19 pandemic. To investigate the impact of the COVID‐19 pandemic, we compared the numbers of surgeries and colonoscopies performed and the patient characteristics between the groups screened before and during the COVID‐19 pandemic. We also evaluated differences in the methods for detecting colorectal cancer performed before and during the COVID‐19 pandemic.

### 2.3 Statistical analysis

Statistical analyses were performed with JMP Pro 14 software (SAS Institute, Cary, NC, USA). Patient characteristics were compared with Fisher’s exact test, the Mann–Whitney test, or the *χ* ^2^ test, as appropriate. Probabilities <0.05 were considered statistically significant.

## 3 RESULTS

### 3.1 Characteristics of institutions

Of the 32 participating institutions, 19 (59.4%) had 251–500 beds and seven (21.9%) had 501–750 beds (Table 1). Half of the institutions performed 51–150 colorectal cancer surgeries per year. A large majority of institutions (81.3%) were partially involved in COVID‐19 care, and 15 institutions (46.9%) had placed restrictions on gastrointestinal surgery, due to the COVID‐19 pandemic.

**TABLE 1:** Characteristics of 32 institutions included in the study

Characteristics	*N* (%)
Number of beds	
≤250	4 (12.5)
251–500	19 (59.4)
501–750	7 (21.9)
≥751	2 (6.3)
Annual number of colorectal cancer surgery	
≤50	7 (21.9)
51–150	16 (50.0)
≥151	9 (28.1)
Hospital involvement in COVID‐19 care	
Partially dedicated	26 (81.3)
Not involved	6 (18.8)
Restrictions on gastrointestinal surgery	
Yes	15 (46.9)
No	17 (53.1)

Data are the number of institutions (%).

**FIGURE 1:**
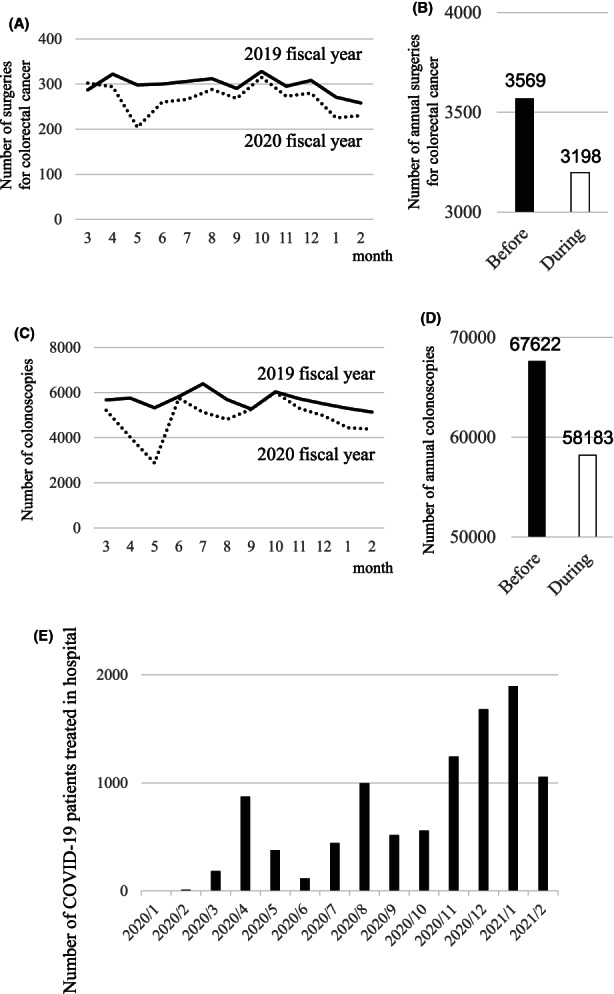
The impact of the COVID‐19 pandemic on surgeries and colonoscopies for colorectal cancer in Japan. (A) Trends in the numbers of surgeries for colorectal cancer performed over 1 y at 32 institutions, before (*solid line*) and during (*dotted line*) the pandemic. (B) Comparison of the total numbers of annual surgeries for colorectal cancer performed before and during the COVID‐19 pandemic. (C) Trend of the number of colonoscopies performed over 1 y at 32 institutions, before (*solid line*) during (*dotted line*) the pandemic. (D) Comparison of the total numbers of annual colonoscopies for colorectal cancer performed before and during the COVID‐19 pandemic. (E) Monthly numbers of patients with COVID‐19 treated in hospital at 32 institutions

During the COVID‐19 pandemic year, the monthly number of surgeries performed for colorectal cancer decreased at our 32 institutions, compared to the year before the pandemic (Figure 1A). In particular, in May 2020, the number decreased by 31.5%, compared to May in the previous year. During the pandemic year, 3198 colorectal cancer surgeries were performed, which represented a reduction of 371 cases, compared to the year before the pandemic (Figure 1B).

The monthly number of colonoscopies performed at all 32 institutions also decreased during the pandemic year compared to the previous year (Figure 1C). In particular, in May a 46% decrease was observed. During the pandemic year, 58 183 colonoscopies were performed, which represented a reduction of 9439 colonoscopies, compared to the previous year (Figure 1D). Increases in the numbers of patients hospitalized with COVID‐19 each month corresponded to increases in the numbers of individuals infected (Figure 1E).

### 3.2 Patient characteristics

This study included 6767 patients who underwent surgery for colorectal cancer between March 2019 and February 2021 at 32 institutions (Table 2). Of these, 5219 (77.1%) patients underwent laparoscopy, 815 (12.0%) underwent open surgery, and 733 (10.8%) underwent robot‐assisted surgery. Additionally, 6505 patients (96.1%) underwent a primary resection of the tumor, 265 (3.9%) required emergency surgery, 486 (7.2%) underwent a multivisceral resection, and 492 (7.3%) had obstructive colorectal cancer. Cancer staging showed that 1732 (25.6%), 1969 (29.1%), 2044 (30.2%), and 1022 (15.1%) patients had stage 0–I, II, III, or IV, respectively.

**TABLE 2:** Characteristics of 6767 patients who underwent surgery for colorectal cancer

Characteristic	*N* (%)
Sex, male/female	3031/3736
Age, y	72 (20–102)
BMI, kg/m^2^	22.0 (7.5–63.9)
ASA‐PS	
1	1133 (16.7)
2	4397 (65.0)
3	1179 (17.4)
4	47 (0.7)
5	11 (0.1)
Tumor location	
Right side	2415 (35.7)
Left side	4352 (64.3)
Depth of tumor invasion	
T0‐T1	1217 (18.0)
T2	827 (12.2)
T3	2895 (42.8)
T4	1828 (27.0)
Lymph node metastasis	
N0	3894 (57.5)
N1	1769 (26.1)
N2	790 (11.7)
N3	314 (4.6)
Distant metastasis	
Positive	1027 (15.2)
Negative	5740 (84.8)
Stage	
0, I	1732 (25.6)
II	1969 (29.1)
III	2039 (30.1)
IV	1027 (15.2)

Data are the number of patients (%) or median (range), unless indicated otherwise.

Abbreviations: BMI, body mass index; ASA‐PS, American Society of Anesthesiologists‐physical status.

### 3.3 Impact of the COVID‐19 pandemic on patient background

Compared to the period before the pandemic, patient characteristics during the COVID‐19 pandemic were not significantly different, in terms of sex, age, or tumor location (Table 3). During the COVID‐19 pandemic, a higher proportion of patients had an ASA‐PS ≥3, compared to the group treated before the COVID‐19 pandemic (19.7% vs 17.0%; *P* = .004). The two groups had similar proportions of lymph node metastasis and distant metastasis, but the proportions of patients with tumor invasion depths ≤T2 (26.2% vs 28.6%; *P* = .025) and clinical stages ≤I (24.2% vs 26.9%; *P* = .011) were significantly smaller during the COVID‐19 pandemic than before the pandemic. The two groups included similar proportions of primary tumor resections, emergency surgeries, multivisceral resections, obstructive colorectal cancers, and chemotherapy or chemoradiotherapy before surgery.

**TABLE 3:** Characteristics of patients with colorectal cancer treated before and during the COVID‐19 pandemic

Characteristic	Before COVID‐19 (*N* = 3569)	During COVID‐19 (*N* = 3198)	*P* ^b^
Sex, male/female	1947/1622	1789/1409	.260
Age, y	72 (20–102)	72 (24–98)	.136^c^
BMI, kg/m^2^	21.9 (7.5–43.4)	22.1 (8.3–63.9)	.049^c^
ASA‐PS			.004
≤2	2963 (83.0%)	2567 (80.3%)	
≥3	606 (17.0%)	631 (19.7%)	
Tumor location			.242
Right side	1297 (36.3%)	1118 (35.0%)	
Left side	2272 (63.7%)	2080 (65.0%)	
Depth of tumor invasion			.025
≤T2	1022 (28.6%)	838 (26.2%)	
≥T3	2547 (71.4%)	2360 (73.8%)	
Lymph node metastasis			.883
Positive	1512 (42.4%)	1361 (42.6%)	
Negative	2057 (57.6%)	1837 (57.4%)	
Distant metastasis			.659
Positive	535 (15.0)	492 (15.4)	
Negative	3034 (85.0)	2706 (84.6)	
Stage			.011
≤I	959 (26.9%)	773 (24.2%)	
≥II	2610 (73.1%)	2425 (75.8%)	
Approach			<.001^d^
Open	449 (12.6%)	366 (11.4%)	
Laparoscopic	2803 (78.5%)	2416 (75.6%)	
Robot‐assisted	317 (8.9%)	416 (13.0%)	
Primary tumor resection	3438 (96.3%)	3067 (95.9%)	.377
Emergency surgery	135 (3.8%)	130 (4.1%)	.572
Multivisceral resection	257 (7.2%)	229 (7.2%)	.962
Obstructive colorectal cancer^a^	267 (7.5%)	225 (7.0%)	.512
Chemotherapy or chemoradiotherapy before surgery	132 (3.7%)	119 (3.7%)	1.000

Data are the median (range) or number (%) of patients, unless indicated otherwise.

^a^ Obstructive colorectal cancer included only cases classified as grade III or higher in the Clavien–Dindo classification system; BMI: body mass index; ASA‐PS: American Society of Anesthesiologists‐physical status.

^b^ *P*‐values were determined with the Fisher’s exact test.

^c^ Mann–Whitney test or

^d^ χ^2^ test.

### 3.4 Impact of the COVID‐19 pandemic on methods of detecting colorectal cancer

We investigated the impact of the COVID‐19 pandemic on how colorectal cancer was detected, due to restrictions placed on colorectal cancer screening during the pandemic. The rate of colorectal cancer detected with fecal occult blood tests was significantly lower during the COVID‐19 pandemic than before the pandemic (26.2% vs 29.6%; *P* = .002) (Table 4). In contrast, the proportions of colorectal cancer detected by abdominal symptoms, by anemia, or accidentally were similar during the two time periods.

**TABLE 4:** Methods of detecting colorectal cancer before and during the COVID‐19 pandemic

Method	Before COVID‐19 *N* (%)	During COVID‐19 *N* (%)	Change, %	*P*
Total				
Fecal occult blood test	1055 (29.6%)	837 (26.2%)	−3.4	.002
Abdominal symptoms	1797 (50.4%)	1658 (51.8%)	1.4	.223
Anemia	520 (14.6%)	514 (16.1%)	1.5	.091
Accidental	290 (8.1%)	240 (7.5%)	−0.6	.365
Institutions with >100 annual colorectal cancer surgeries				
Fecal occult blood test	794 (30.2%)	624 (26.6%)	−3.6	.006
Abdominal symptoms	1322 (50.3%)	1218 (52.0%)	1.7	.233
Anemia	326 (12.4%)	331 (14.1%)	1.7	.078
Accidental	190 (7.2%)	160 (6.8%)	−0.4	.617
Institutions with ≤100 annual colorectal cancer surgeries				
Fecal occult blood test	261 (27.8%)	213 (24.9%)	−2.9	.180
Abdominal symptoms	475 (50.6%)	440 (51.5%)	0.9	.741
Anemia	194 (20.7%)	183 (21.4%)	0.7	.728
Accidental	100 (10.7%)	80 (9.4%)	−1.3	.387

*P*‐values were determined with the Fisher’s exact test.

In general, the number of colorectal cancer cases detected by screening tends to be higher in facilities with larger numbers of colorectal cancer surgeries. Therefore, we investigated whether the change in colorectal cancer detection rates before and during the COVID‐19 pandemic corresponded to the annual numbers of surgeries performed at the 32 institutions. In institutions with more than 100 annual colorectal cancer surgeries, the rate of colorectal cancer detected by fecal occult blood test during the COVID‐19 pandemic was significantly lower than that before the COVID‐19 pandemic (26.6% vs 30.2%; *P* = .006). Conversely, in institutions with less than 100 annual colorectal cancer surgeries, the rates of fecal occult blood test detection did not change significantly during the pandemic. The rates of using other colorectal cancer detection methods (ie, abdominal symptoms, anemia, or accidental) did not significantly change during the pandemic, whether hospitals had high or low annual numbers of colorectal cancer surgeries.

### 3.5 Impact of COVID‐19 on colorectal cancer treatments

The patients treated during the COVID‐19 pandemic tended to be in worse condition than those treated before the pandemic, but only in institutions with more than 100 colorectal cancer surgeries per year (ASA‐PS ≥3: 20.6% vs 17.2%; *P* = .002) (Table 5). In these institutions, during the pandemic the proportion of tumor invasion depths ≤T2 decreased (26.8% vs 29.6%; *P* = .030), and the proportion of patients with clinical stage cancers ≤I decreased (24.3% vs 27.8%; *P* = .006), compared to the proportions observed in the prior year. On the other hand, these trends were not observed in institutions with fewer than 100 annual colorectal cancer surgeries (Table 6).

**TABLE 5:** Characteristics of patients with colorectal cancer treated before or during the COVID‐19 pandemic in institutions with more than 100 colorectal cancer surgeries per year

Characteristic	Before COVID‐19 (*N* = 2631)	During COVID‐19 (*N* = 2344)	*P* ^b^
Sex, male/female	1449/1182	1309/1035	.587
Age, y	71 (20–97)	72 (24–98)	.010^c^
BMI, kg/m^2^	21.9 (7.5–43.4)	22.1 (8.3–63.9)	.017^c^
ASA‐PS			.002
≤2	2179 (82.8%)	1861 (79.4%)	
≥3	452 (17.2%)	483 (20.6%)	
Tumor location			.455
Right side	921 (35.0%)	796 (34.0%)	
Left side	1710 (65.0%)	1548 (66.0%)	
Depth of tumor invasion			.030
≤T2	779 (29.6%)	628 (26.8%)	
≥T3	1852 (70.4%)	1716 (73.2%)	
Lymph node metastasis			.818
Positive	1101 (41.9%)	989 (42.2%)	
Negative	1530 (58.1%)	1355 (57.8%)	
Distant metastasis			.811
Positive	393 (14.9%)	344 (14.7%)	
Negative	2238 (85.1%)	2000 (85.3%)	
Stage			.006
≤I	732 (27.8%)	570 (24.3%)	
≥II	1899 (72.2%)	1774 (75.7%)	
Approach			<.001^d^
Open	327 (12.4%)	263 (11.2%)	
Laparoscopic	1987 (75.5%)	1669 (71.2%)	
Robot‐assisted	317 (12.1%)	412 (17.6%)	
Primary tumor resection	2547 (96.8%)	2266 (96.7%)	.811
Emergency surgery	102 (3.9%)	94 (4.0%)	.827
Multivisceral resection	210 (8.0%)	173 (7.4%)	.456
Obstructive colorectal cancer^a^	186 (7.1%)	138 (5.9%)	.095

Data are the median (range) or number (%) of patients, unless otherwise indicated.

^a^ Obstructive colorectal cancer included only cases classified as grade III or higher in the Clavien–Dindo classification system; BMI: body mass index; ASA‐PS: American Society of Anesthesiologists‐physical status.

^b^ *P*‐values were determined with the Fisher’s exact test.

^c^ Mann–Whitney test, or

^d^ *χ* ^2^ test.

**Table 6:** Characteristics of patients with colorectal cancer treated before or during the COVID‐19 pandemic in institutions with less than 100 annual colorectal cancer surgeries

Characteristic	Before COVID‐19 (*N* = 938)	During COVID‐19 (*N* = 854)	*P* ^b^
Sex, male/female	498/440	480/374	.200
Age, y	73 (29–97)	74 (31–102)	.149^c^
BMI, kg/m^2^	21.9 (10.1–39.2)	22.0 (12.1–39.5)	.876^c^
ASA‐PS			.614
≤2	784 (83.6%)	706 (82.7%)	
≥3	154 (16.4%)	148 (17.3%)	
Tumor location			.309
Right side	376 (40.1%)	322 (37.7%)	
Left side	562 (59.9%)	532 (62.3%)	
Depth of tumor invasion			.674
≤T2	268 (28.6%)	236 (27.6%)	
≥T3	670 (71.4%)	618 (72.4%)	
Lymph node metastasis			.924
Positive	411 (43.8%)	372 (43.6%)	
Negative	527 (56.2%)	482 (56.4%)	
Distant metastasis			.222
Positive	142 (15.1%)	148 (17.3%)	
Negative	796 (84.9%)	706 (82.7%)	
Stage			.868
≤I	227 (24.2%)	203 (23.8%)	
≥II	711 (75.8%)	651 (76.2%)	
Approach			.094^d^
Open	122 (13.0%)	103 (12.1%)	
Laparoscopic	816 (87.0%)	747 (87.5%)	
Robot‐assisted	0 (0%)	4 (0.5%)	
Primary tumor resection	891 (95.0%)	801 (93.8%)	.303
Emergency surgery	33 (3.5%)	36 (4.2%)	.463
Multivisceral resection	47 (5.0%)	56 (6.6%)	.186
Obstructive colorectal cancer^a^	81 (8.6%)	87 (10.2%)	.292

Data are the median (range) or number (%) of patients, unless otherwise indicated.

^a^ Obstructive colorectal cancer included only cases classified as grade III or higher in the Clavien–Dindo classification system; BMI: body mass index; ASA‐PS: American Society of Anesthesiologists‐physical status.

^b^ *P*‐values were determined with the Fisher’s exact test.

^c^ Mann–Whitney test, or

^d^ *χ* ^2^ test.

## 4 DISCUSSION

The COVID‐19 pandemic has disrupted healthcare systems around the world, resulting in fewer endoscopies performed, and fewer cancers detected.^15^, ^16^ A previous retrospective study of two Japanese institutions reported also that the number of colorectal cancer diagnoses had decreased, due to the COVID‐19 pandemic.^17^ We covered a large number of institutions, by incorporating 32 institutions affiliated with Osaka University, collected detailed information on each institution, and examined the methods used for detecting colorectal cancer for each patient. In fact, these 32 institutions treat about one‐third of the 10 000 colorectal cancer cases treated annually in Osaka, the third largest city in Japan. To our knowledge, this study was the first large‐scale multicenter study to suggest that the COVID‐19 pandemic reduced the number of colonoscopies and surgeries performed for colorectal cancer and hindered the detection of asymptomatic early‐stage cancers, and that these trends were more significant in larger institutions, enlightening colorectal surgeons that the impact of COVID‐19 varied by the hospital size, even if the region was the same.

We found that, due to the COVID‐19 pandemic, the annual number of colonoscopies performed in our region dropped significantly, from 67 622 to 58 183. The Japan Gastroenterological Endoscopy Society recommended that the screening and surveillance of nonurgent gastrointestinal endoscopies should be postponed under a declared state of emergency. However, they also recommended that, in the absence of a declared state of emergency, regular endoscopic examinations should be performed as usual, including for asymptomatic patients that were not clinically suspected of having COVID‐19, provided that appropriate triage and reliable infection prevention measures were implemented.^11^ Indeed, it is important to consider the risk of COVID‐19 infections in gastrointestinal endoscopy; nevertheless, we should also consider that some patients can be saved with gastrointestinal endoscopy. During the COVID‐19 pandemic, a higher proportion of patients had an ASA‐PS ≥3, which might be explained by the possibility that they had serious systemic diseases and were therefore more likely to visit a hospital and be screened for the detection of colorectal cancer.

The Japanese Surgical Society also proposed surgical triage similar to that of the American College of Surgeons; they recommended that scheduled surgeries for nonurgent diseases should be postponed, in principle; surgeries for nonfatal, but potentially serious diseases should be postponed, when possible; and surgeries for potentially fatal diseases should be performed immediately and carefully, with adequate infection control measures.^18^, ^19^ During the COVID‐19 pandemic, all 32 institutions in this study performed surgery based on this surgical triage proposed by the Japanese Surgical Society. About half of our institutions had restrictions on gastrointestinal surgery, of which almost all were for surgery for benign diseases. Although it might be acceptable to postpone early‐stage colorectal cancer surgeries, we tried to perform surgery without delay because early‐stage cancer could also be potentially fatal. The national population‐based study in England reported that the average number of elective surgeries for colorectal cancer dropped sharply during the pandemic, from 386 to 214 per week.^20^ In the present study, the number of colorectal cancer surgeries (particularly early‐stage) also declined during the pandemic, as well as the number of lower‐gastrointestinal endoscopies and colorectal cancers diagnosed with fecal occult blood tests, suggesting that the pandemic prevented early cancer detection. Although in this study the proportions of primary tumor resections, emergency surgeries, other organ resections, and obstructive colorectal cancers did not change significantly during the COVID‐19 pandemic, the incidences of detecting colorectal cancer obstruction or perforation at an advanced stage may increase in the future. Therefore, further study is needed to determine when the overlooked colorectal cancers might be detected and surgically treated. And it is important to recognize that COVID‐19 affects diagnosis and surgery, not only in colorectal cancer, but also in other cancers for which postponement of surgery has been reported.^21^

A previous study showed that, among patients with positive fecal occult blood tests, follow‐ups that were delayed for more than 10 mo were associated with a higher risk of colorectal cancer and a more advanced stage at diagnosis, compared to follow‐ups with colonoscopy between 8 and 30 d.^22^ During the COVID‐19 pandemic, delaying surgery for 12 weeks for solid cancers, including colon, breast, and lung cancers, could reduce survival.^23^ Accordingly, the decline and delays we observed in colonoscopies and surgeries performed for colorectal cancer during the COVID‐19 pandemic may have a negative impact on the prognosis. Future studies will need to assess the impact of the COVID‐19 pandemic on the prognosis of patients with colorectal cancer.

The main limitation of this study was that it was conducted for only 2 y (2019 and 2020 fiscal years). Evaluations for longer observation periods may reveal a more accurate picture of the impact of COVID‐19. Another limitation of this study was that, although we analyzed data from 32 institutions, the population was mainly in Osaka. Thus, the generalizability of the results may be limited. A larger analysis, based on the National Clinical Database of Japan, might allow a more detailed study.^24^

In our study the tendency that the number of early colorectal cancer surgeries and colorectal cancers diagnosed with fecal occult blood tests declined was more pronounced in larger facilities. This might be due to the fact that institutions with more than 100 annual colorectal cancer surgeries tended to have larger proportions of colorectal cancer diagnoses based on positive fecal occult blood tests and larger numbers of Stage I or lower colorectal cancer cases, compared to institutions with less than 100 annual colorectal cancer surgeries before the pandemic.

In conclusion, our findings suggested that it is important to consider the impact of COVID‐19 from a broader perspective, because the impact can be different, depending on the characteristics of each facility and region. Moreover, understanding the different impacts on different institutions could facilitate collaboration among regions, guided by the national healthcare system, to provide appropriate care to all patients.

## DISCLOSURE

Conflict of interest: Tsunekazu Mizushima and Yuichiro Doki are members of the Editorial Board of *Annals of Gastroenterological Surgery*.

Ethical approval: The study was approved by the Institutional Review Board for Studies in Humans at Osaka University (approval number: 20527) and at each institution.

Informed consent: Informed consent was waived owing to the retrospective nature of the study. The opt‐out recruitment method was applied to all patients, with an opportunity to decline to participate.

Registry and the registration no. of the study/trial: N/A.

Animal studies: N/A.
